# Receptor tyrosine kinase profiling of ischemic heart identifies ROR1 as a potential therapeutic target

**DOI:** 10.1186/s12872-018-0933-y

**Published:** 2018-10-20

**Authors:** Juho Heliste, Anne Jokilammi, Ilkka Paatero, Deepankar Chakroborty, Christoffer Stark, Timo Savunen, Maria Laaksonen, Klaus Elenius

**Affiliations:** 1 Institute of Biomedicine, University of Turku, Kiinamyllynkatu 10, FIN-20520 Turku, Finland; 2 Turku Doctoral Programme of Molecular Medicine, University of Turku, Turku, Finland; 3 Institute for Molecular Medicine Finland, University of Helsinki, Helsinki, Finland; 4 Turku Centre for Biotechnology, University of Turku and Åbo Akademi University, Turku, Finland; 5 Research Center of Applied and Preventive Cardiovascular Medicine, University of Turku, Turku, Finland; 6 Medisapiens Ltd., Helsinki, Finland; 7 Medicity Research Laboratories, University of Turku, Turku, Finland; 8 Department of Oncology, Turku University Hospital, Turku, Finland

**Keywords:** Hypoxia, Ischemic cardiomyopathy, Myocardial ischemia, Myocardial infarction, Receptor tyrosine kinase

## Abstract

**Background:**

Receptor tyrosine kinases (RTK) are potential targets for the treatment of ischemic heart disease. The human RTK family consists of 55 members, most of which have not yet been characterized for expression or activity in the ischemic heart.

**Methods:**

RTK gene expression was analyzed from human heart samples representing healthy tissue, acute myocardial infarction or ischemic cardiomyopathy. As an experimental model, pig heart with ischemia-reperfusion injury, caused by cardiopulmonary bypass, was used, from which phosphorylation status of RTKs was assessed with a phospho-RTK array. Expression and function of one RTK, ROR1, was further validated in pig tissue samples, and in HL-1 cardiomyocytes and H9c2 cardiomyoblasts, exposed to hypoxia and reoxygenation. ROR1 protein level was analyzed by Western blotting. Cell viability after ROR1 siRNA knockdown or activation with Wnt-5a ligand was assessed by MTT assays.

**Results:**

In addition to previously characterized RTKs, a group of novel active and regulated RTKs was detected in the ischemic heart. ROR1 was the most significantly upregulated RTK in human ischemic cardiomyopathy. However, ROR1 phosphorylation was suppressed in the pig model of ischemia-reperfusion and ROR1 phosphorylation and expression were down-regulated in HL-1 cardiomyocytes subjected to short-term hypoxia in vitro. ROR1 expression in the pig heart was confirmed on protein and mRNA level. Functionally, ROR1 activity was associated with reduced viability of HL-1 cardiomyocytes in both normoxia and during hypoxia-reoxygenation.

**Conclusions:**

Several novel RTKs were found to be regulated in expression or activity in ischemic heart. ROR1 was one of the most significantly regulated RTKs. The in vitro findings suggest a role for ROR1 as a potential target for the treatment of ischemic heart injury.

**Electronic supplementary material:**

The online version of this article (10.1186/s12872-018-0933-y) contains supplementary material, which is available to authorized users.

## Background

Ischemic heart disease is the leading cause of death globally [1]. In the case of an acute myocardial infarction, myocardial damage caused by ischemia is exacerbated by oxygenized blood returning to the heart at reperfusion [2]. Approaches to treat infarction should both promote reperfusion and protect myocardium from the detrimental effects of ischemia and reperfusion.

Receptor tyrosine kinases (RTK) are cell surface receptors that mediate cellular survival, proliferation, and migration. A few RTKs have been shown to be necessary for development of the heart in gene-modified mouse models. Such examples include *Erbb2* [3, 4], *Erbb4* [5], *Ror1* [6], and *Ror2* [6, 7]. Understanding of the regulation of RTK activity and expression in ischemic heart is limited to few receptors. Expression of EGFR and ERBB2 have been demonstrated to be regulated in infarcted human heart [8, 9], and alterations in EGFR, ERBB2, ERBB4, VEGFR1, VEGFR2, IGF1R, and INSR signaling have been observed in experimental ischemia-reperfusion models [8, 10–12].

Few RTKs have been investigated as targets for the treatment of experimental ischemia-reperfusion injury. Induction of constitutively active ERBB2 after infarction causes myocardial regeneration in mice [13]. Activating ERBB4 with its ligand neuregulin-1 reduces scar size in mouse [14] and rat [15] infarction models. Activation of INSR by insulin infusion during reperfusion has been shown to reduce infarction size in an ischemia-reperfusion rat heart model in Langendorff perfusion system [16]. Moreover, glucose-insulin-potassium infusion has been tested in clinical trials as a myocardial infarction treatment with mixed results [17].

Here, we used an in silico expression analysis and a phosphoarray analysis of an in vivo pig ischemia-reperfusion injury model to screen for changes in the expression and activity of RTKs in normal vs. ischemic heart. ROR1 was identified as a receptor demonstrating activity in both screens. We show that ROR1 was expressed in human heart, in pig myocardium and in cultured mouse cardiomyocytes and rat cardiomyoblasts. In cardiomyocytes in vitro, both ROR1 expression and phosphorylation were downregulated by hypoxia. We also demonstrate that ROR1 knockdown enhanced, and treatment with its ligand, Wnt-5a, reduced the viability of cardiomyocytes. These findings suggest that ROR1 signaling may suppress survival of cardiomyocytes and that ROR1 could be further tested as a potential treatment target for the myocardial ischemic injury.

## Methods

### In silico transcriptomics

Affymetrix gene expression data from IST Online database (ist.medisapiens.com; Medisapiens Ltd.) were analyzed to characterize RTK expression in samples representing healthy heart (*n* = 62), acute myocardial infarction (*n* = 12) or ischemic cardiomyopathy (*n* = 63). Out of the 55 RTKs listed by HUGO Gene Nomenclature Committee, data were available for 49 genes in acute myocardial infarction samples and for 52 genes in ischemic cardiomyopathy samples (Additional file 1). Data were normalized by array-generation-based gene centering method [18] and log2-transformed. Expression levels of RTKs demonstrating statistically significant differences in two-group comparisons were visualized as box plots and heatmaps using Pretty Heatmaps package (pheatmap) [19] in RStudio [20].

### Pig model of heart ischemia-reperfusion injury

Animal experiments were approved by the Laboratory Animal Care and Use Committee of the State Provincial Office of Southern Finland (license number: ESAVI/1167/04.10.03/2011). The landrace pig myocardial samples (*n* = 7) were a kind gift from Drs. Christoffer Stark and Timo Savunen. Experimental procedure has been described in detail earlier [21]. Pigs weighed 29–43 kg. Myocardial ischemia-reperfusion injury was produced by exposing anesthetized pigs (*n* = 4) to cardiopulmonary bypass with aortic cross-clamping and cardioplegic arrest for 60 min, causing global myocardial ischemia. A pediatric membrane oxygenator (Dideco 905 Eos, Dideco) was used for the bypass. Procedures were performed in the laboratory of Research Center of Applied and Preventive Cardiovascular Medicine, University of Turku, Turku, Finland. For pre-anesthesia, an intramuscular injection of 100 mg xylazin (Rompun vet, Bayer Animal Health GmbH) and 25 mg midazolam (Midazolam Hameln, Hameln pharmaceuticals GmbH) was used. For anesthesia, 20 mg boluses of propofol (PropofolLipuro, B. Braun Melsungen AG) and 150 μg phentanyl (Fentanyl-Hameln, Hameln pharmaceuticals GmbH) were administered via a cannulated ear vein, and pigs were intubated and connected to a respirator (Dräger Oxylog 3000, Drägerwerk AG), the respiratory rate set to 18–22 times/min with a tidal volume of 8–10 ml/kg using 40% oxygen. Continuous infusion of propofol 15–30 mg/kg/h, phentanyl 1.5 μg/kg/h and midazolam 100 μg/kg/h was used to maintain anesthesia. A right sided thoracotomy was performed and the ascending aorta and right atrium cannulated for the bypass. 500 ml of cold (10 °C) Modified St Thomas Hospital No II cardioplegia was used to protect the hearts during the bypass, administered via a cannula to the aortic root at the time of cross-clamping and 30 min later. Antibiotic prophylaxis (Cefuroxime 750 mg, Orion Pharma) was given preoperatively and then every 8 h. 10,000 IU of heparin (Heparin, LEO Pharma) was administered as a bolus before cannulation of the heart and this was repeated every 30 min during extracorporeal circulation. 14,000 IU of protamine sulphate (Protamin, LEO Pharma) was used to neutralize the heparin. For thrombosis prophylaxis, 20 mg of enoxaparin (Klexane, Sanofi) was administered 1 and 12 h after the surgery. 100–150 mg of lidocaine (Lidocain, Orion Pharmaceuticals) and 150–225 mg of amiodarone (Cordarone, Sanofi) were used for rhythm disorders, and 5 mg boluses of ephedrine (Efedrin, Stragen Nordic) and noradrenaline infusion (80–160 μg/h) (Noradrenalin Hospira, Hospira) were used for post-operative hemodynamic support, when needed. For post-operative analgesia, 50 mg of bupivacaine (Bicain, Orion Pharmaceuticals) was infiltrated to the wound. For monitoring of adequate ventilation and perfusion, blood gases (i-STAT, Abbott Laboratories), invasive central venous pressure, ECG and oxygen saturation were followed throughout the procedure.

After the 60-min aortic cross-clamping, hearts were reperfused and the pigs were maintained anesthetized and mechanically ventilated for 29–31 h before sacrification with intravenous injection of potassium chloride. Control samples (*n* = 3) were obtained from pigs used as blood donors for priming of the heart-lung machine. The control pigs underwent the same anesthetic protocol as the treatment group. Transmural left ventricle samples, collected after the sacrification, were snap-frozen and stored at − 80 °C. Troponin T levels were measured from plasma samples of ischemia-reperfusion-injured pigs, collected at the baseline and 6 and 24 h after reperfusion, by the laboratory of the Turku University Central Hospital using electrochemiluminescence immunoassay (Elecsys Troponin T high sensitive, Roche). Formalin-fixed, paraffin-embedded tissue samples were stained with hematoxylin and eosin and imaged with Zeiss AxioImager M1 microscope.

### Phosphoarray analysis of RTK phosphorylation

Pig myocardial samples (280 to 460 mg) were homogenized and analyzed for phosphorylation status of 49 RTKs using the Proteome Profiler Human Phospho-RTK Array Kit (R&D Systems). Five hundred μg of protein was analyzed per sample. Receptors included in the analysis are listed in Additional file 2 B.

Array blot images were quantified by densitometry with NIH ImageJ v1.50i software. Intensity values were normalized by dividing each dot’s intensity with the sum of intensities of the whole array, allowing comparison between different samples. Data were scaled to interval 0–1 by dividing all values with the highest value. Normalized values were visualized as a bargraph (mean (SD)) and as a heatmap with Pretty Heatmaps package [19]. Receptors with at least two quantifiable results in both sample groups were included. Receptors were clustered using maximum distance method.

### Cell culture and Wnt-5a ligand treatment

HL-1 mouse atrial cardiomyocytes were a kind gift from Dr. Pasi Tavi (University of Eastern Finland). HL-1 cells were maintained in Claycomb medium (Sigma) supplemented with 10% FBS, 0.1 mM norepinephrine, 50 U/ml penicillin, 50 U/ml streptomycin, and 2 mM UltraGlutamine (Lonza). Culture plates were coated at 37 °C with a solution containing 0.02% gelatin and 10 μg/ml fibronectin. Seeding densities were 140,000 cells/6-well plate well, 100,000 cells/12-well plate well, and 5,000 cells/96-well plate well. H9c2 rat cardiomyoblasts were purchased from ATCC and maintained in DMEM with 1.5 g/l NaHCO_3_, supplemented with 10% FBS, 50 U/ml penicillin, 50 U/ml streptomycin, and 2 mM UltraGlutamine. Seeding density was 100,000 cells/6-well plate well. Cells were routinely checked for mycoplasma infection using MycoAlert Mycoplasma Detection Kit (Lonza). For hypoxia-reoxygenation experiments, the cells were cultured in 1% O_2_ in a hypoxic work station (InVivo_2_, Ruskinn Technology Ltd.) for the indicated periods of time, and returned to normal cell incubator (21% O_2_) for reoxygenation. In ROR1 ligand activation experiments, 200–400 ng/ml of recombinant human/mouse Wnt-5a (R&D Systems) was added to medium at the time of plating (for MTT assays) or 24 h after plating for 30–60 min (for Western analyses).

### RNA interference

One day after plating, HL-1 cells were transfected with siRNAs (Qiagen) targeting *ROR1* (siRNA #1, SI01404655; siRNA #2, SI01404662), or *ROR2* (siRNA #1, SI01404683; siRNA #2, SI01404690) or with AllStars Negative Control siRNA at a concentration of 100 nM, using Lipofectamine 2000 (Invitrogen). Immediately prior to transfection, medium was changed to antibiotic- and norepinephrine-free Claycomb medium. Four to six hours after transfection, medium was replaced with antibiotic-free, norepinephrine-supplemented Claycomb medium.

### RNA extraction and real-time RT-PCR

RNA was extracted from pig myocardium samples using TRIsure reagent (Bioline). Samples were treated with 10 units of DNAse I (Roche). cDNA was synthesized with SensiFAST cDNA Synthesis Kit (Bioline), using 1 μg of total RNA/sample. Real-time RT-PCR was carried out using QuantStudio 12 K Flex Real-Time PCR System thermal cycler (Thermo Fisher Scientific). For PCR reactions, 5 μl of TaqMan Universal Master Mix II (Thermo Fisher Scientific) and 10 ng of template cDNA were used in a reaction volume of 10 μl. Primer concentrations were 0.3 μM and probe concentration 0.1 μM. Primers were acquired from Eurofins Genomics and probes from Universal Probe Library (Roche). *GAPDH* was used as the reference gene [8]. *ROR1* was analyzed using the primers 5’-GCGGCTCGCAATATTCTC-3′ and 5’-GAAAGCCCAAGGTCTGAAATC-3′, and the probe #108. *GAPDH* was analyzed using the primers 5’-ACAGACAGCCGTGTGTTCC-3′ and 5’-ACCTTCACCATCGTGTCTCA-3′, and the probe #28.

### Western blotting and immunoprecipitation

Cells were lysed with lysis buffer [22] supplemented with Pierce Protease Inhibitor Mini Tablets (Thermo Fisher Scientific). Lysates were centrifuged for 15 min at 16,000 g and the supernatants were collected. Snap-frozen pig heart tissue samples were dissolved in ice-cold Lysis Buffer 17 supplied with the Proteome Profiler Human Phospho-RTK Array Kit. Samples were separated on 8–10% polyacrylamide gels. Protein amounts loaded on the gel were 20–35 μg for cell samples and 100 μg for pig tissue samples. Separated samples were transferred to nitrocellulose membrane which was blocked with 5% non-fat milk or bovine serum albumin in 10 mM Tris-HCl (pH 7.4), 150 mM NaCl and 0.05% Tween-20 (blocking solution) for 1 h at room temperature. Membranes were incubated with primary antibodies overnight at 4 °C in the blocking solution. The following primary antibodies and dilutions were used: anti-ROR1 (sc-83033 and sc-130386, Santa Cruz Biotechnology; 1:250 and 1:125, respectively), anti-actin (sc-1616, Santa Cruz Biotechnology; 1:1,000), anti-α-tubulin (sc-5546, Santa Cruz Biotechnology; 1:1,000), anti-phospho-Akt (#4060, Cell Signaling Technology; 1:1,000), anti-Akt (#9272, Cell Signaling Technology; 1:1,000), anti-phospho-p38 (#9211, Cell Signaling Technology; 1:500), anti-p38 (#9212, Cell Signaling Technology; 1:500), anti-phospho-tyrosine (4 g10, Upstate; 1:500), and anti-GAPDH (G8795, Sigma-Aldrich; 1:1,000). Incubation with secondary HRP-conjugated antibodies (Santa Cruz Biotechnology; 1:5,000) or IRDye secondary antibodies (LI-COR, 1:10,000) was carried out for 1 h at room temperature in the blocking solution. The immunosignals were visualized with WesternBright ECL HRP substrate reagent (Advansta) and imaged with ImageQuant LAS 4000 (GE Healthcare Life Sciences) or with Odyssey imaging system (LI-COR). Densitometric analysis of the Western signals was carried out with ImageJ.

For immunoprecipitation analyses, approximately 2 mg of total protein from HL-1 cell lysates was incubated overnight at 4 °C with 4 μg of mouse monoclonal anti-ROR1 antibody (sc-130386, Santa Cruz Biotechnology). Immunoprecipitated lysates were incubated with Protein G PLUS-Agarose beads (sc-2002, Santa Cruz Biotechnology) for one hour at 4 °C. After washing the beads three times with lysis buffer without protease inhibitors, samples from the supernatants were loaded onto 10% polyacrylamide gels for subsequent Western analysis.

### MTT cell viability assay

CellTiter 96 Non-Radioactive Cell Proliferation Assay (MTT) (Promega) was used to measure viability of HL-1 cells. The assay was performed on 96-well plates with plating density of 5,000 cells/well. Before addition of the MTT dye solution, the culture medium was replaced by norepinephrine-free Claycomb medium (100 μl/well). Wells only containing the medium were used for background subtraction. Absorbances at 570 nm were detected with EnSight Multimode Plate Reader (PerkinElmer) and results were normalized to untreated control sample level.

### Protein sequence alignment

Human and pig RTK protein sequences from UniProt database were aligned using EMBOSS Needle tool for global alignments and EMBOSS Water tool for local alignments by the European Bioinformatics Institute (http://www.ebi.ac.uk/Tools/psa/) [23]. The longest pig sequences available were used.

### Statistical analyses

For in silico transcriptomics analyses, Student’s t-test was used to assess the significance of differences between the expression levels of an RTK between sample groups. Correction for multiple testing was performed using false discovery rate (FDR) method [24]. Genes with significant differences in expression were selected for visualizations. Two-tailed Student’s t-test was used for testing the significance of differences in phosphorylation array data (for RTKs with at least three quantifiable results in both treatments) and in expression levels in Western analyses. For in vitro experiments, data are represented as box plots depicting median (black horizontal line), first and third quartile (box) and the range of the data (whiskers). Multiple group comparisons were performed with Kruskal-Wallis test with Dunn’s post-hoc test using FDR method for *P*-value adjustments. *P*-values < 0.05 were considered significant. Analyses were performed with RStudio.

## Results

### Regulation of RTK expression in ischemic human heart

**Fig. 1:**
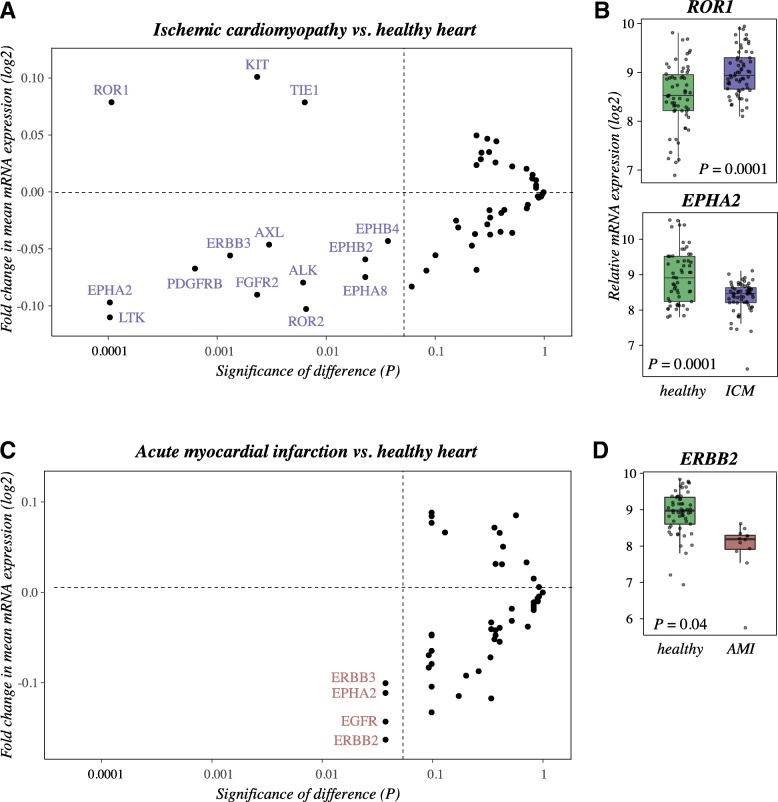
Regulation of RTK expression in acute myocardial infarction and ischemic cardiomyopathy*.* Messenger RNA expression of 52 RTKs in human tissue samples representing healthy heart (*n* = 62), ischemic cardiomyopathy (*n* = 63), or acute myocardial infarction (*n* = 12) was analyzed in silico using IST Online database. **a** A dotplot presentation of FDR-corrected *P* values vs. the fold changes of RTK expression levels between samples representing ischemic cardiomyopathy or healthy heart. **b** A box plot presentation of expression levels of the most significantly up- or downregulated RTKs in ischemic cardiomyopathy vs. healthy heart. **c** A dotplot presentation of FDR-corrected *P* values vs. the fold changes of RTK expression levels between samples representing acute myocardial infarction or healthy heart. **d** A box plot presentation of expression level of the most significantly downregulated RTK in acute myocardial infarction vs. healthy heart. In A and C, the fold changes of means were calculated from log2-transformed Affymetrix expression values (arbitrary units). Vertical grey lines depict the threshold *P* = 0.05. RTKs not demonstrating significant (*P* < 0.05) changes in expression are only depicted by dots without labels

Expression of human RTKs in healthy heart, heart with ischemic cardiomyopathy and heart with acute myocardial infarction was analyzed in silico using the IST Online database. Comparison of healthy samples to ischemic cardiopathy samples revealed a set of 14 receptors that were significantly differently expressed at the mRNA level (Fig. 1a). The most significantly up- and downregulated RTKs were *ROR1* and *EPHA2*, respectively, when ischemic cardiomyopathy was compared to normal heart (Fig. 1b). Interestingly, another ROR family member, *ROR2*, was downregulated in the ischemic cardiomyopathy samples (Fig. 1a). When comparing healthy hearts with hearts representing acute myocardial infarction, significant downregulation of *EGFR, ERBB2, ERBB3*, as well as *EPHA2*, was discovered in the infarcted heart, *ERBB2* demonstrating the greatest difference in expression (Fig. 1c and d). Statistical test values and differences in expression are shown in Additional file 1 and heatmaps generated from the data in Additional file 3.

### RTK phosphorylation in experimental pig ischemia-reperfusion model

**Fig. 2:**
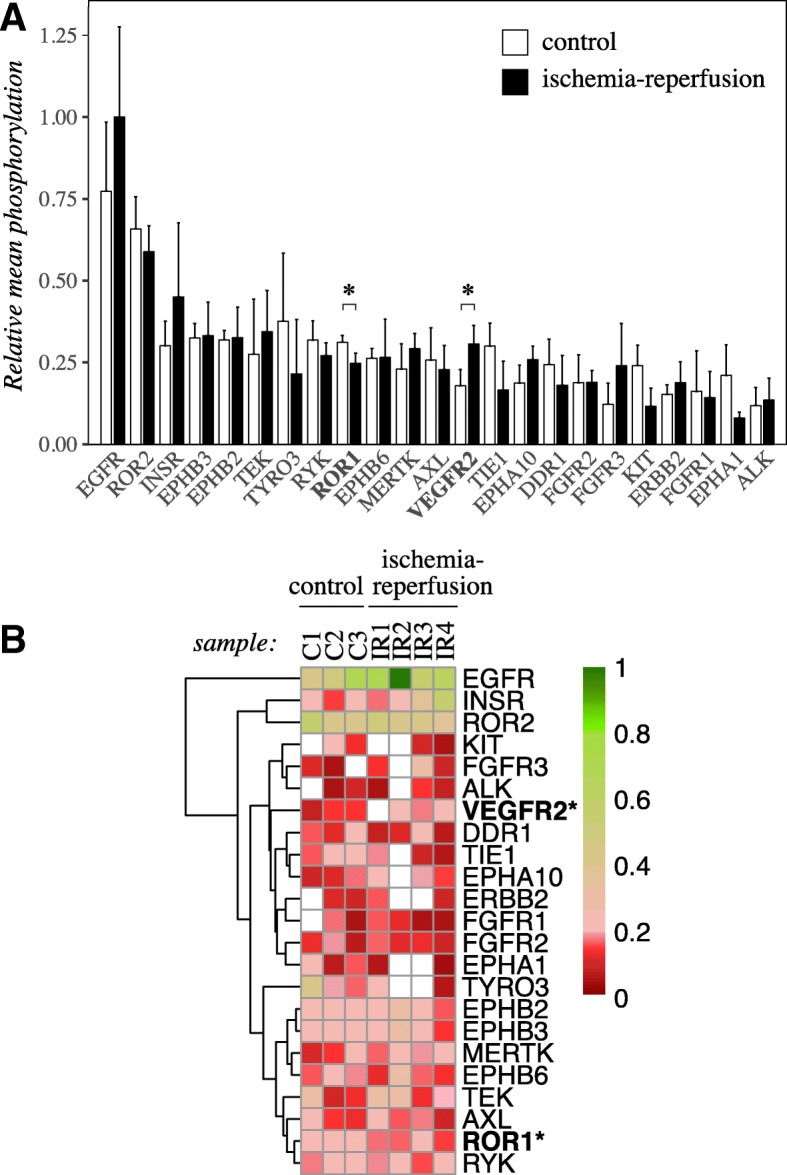
RTK phosphorylation in a pig model of ischemia-reperfusion injury*.* A phospho-RTK array analysis addressing the phosphorylation of 49 RTKs was carried out for heart samples from a pig model of ischemia-reperfusion injury. Control pigs underwent the same anesthetic protocol as the ischema-reperfusion-injured pigs. **a** The intensity of the dots in the phospho-array were quantified by densitometry and normalized to each array’s sum of intensities. The resulting values were scaled according to the highest value set to one. Normalized phosphorylation values of each receptor with at least two non-zero results per each treatment are shown (mean + SD). **b** The same set of normalized phospho-RTK dot intensities were visualized as a heatmap. Receptors with at least two samples with non-zero results per each treatment were included, and were clustered using the maximum distance method. White tiles depict missing data for few samples. Asterisk indicates significant difference in phosphorylation (*P* < 0.05) for comparison of control samples to ischemia-reperfusion samples

To experimentally address RTK activation in the ischemic heart, an ischemia-reperfusion model in pig, a relevant model animal close to human as a large mammal, was used. Pigs were subjected to ischemia-reperfusion injury by cardiopulmonary bypass and reperfusion, followed by phospho-RTK array analysis of myocardial samples. Increased amount of troponin T in the plasma (Additional file 4 A) and thinned cardiomyocytes with condensed nuclei as demonstrated by histological analysis (Additional file 4 B) indicated ischemic injury. The phosphoarray analysis was carried out after approximately 30 h of reperfusion. While the observed phosphorylation-specific signal was relatively weak (possibly due to loss of phosphosignal during sample preparation and/or low cross-reactivity of the array antibodies between human and pig epitopes) (Additional file 2 A), the data indicated the presence of 23 activated RTKs in the heart. As expected, based on publications about RTK activity in the heart [8–12], the array identified phosphorylated EGFR, INSR, VEGFR2, and ERBB2 in both ischemic (*n* = 4) and control samples (*n* = 3) (Fig. 2a). Phosphorylation of a group of receptors, that to our knowledge have not previously been shown to be active or regulated in ischemic heart, was also detected. This group included both ROR receptors ROR1 and ROR2; the EPH receptors EPHB2, EPHB3, EPHB6, and EPHA10; TYRO3; RYK; FGFR3 and ALK (Fig. 2a).

Densitometric quantitation of the data indicated highest activity for EGFR, ROR2, INSR, EPHB3, EPHB2, TEK, TYRO3, RYK, ROR1 and EPHB6 (phosphosignal exceeding the 75th percentile of mean phosphorylation levels of all RTKs in all samples). Phosphorylation of ROR1 was significantly downregulated (*P* = 0.022), and phosphorylation of VEGFR2 significantly upregulated (*P* = 0.043), in ischemia-reperfusion-injured hearts. Unsupervised clustering of the RTKs on a heatmap grouped highly active EGFR, INSR and ROR2 together. ROR1 and RYK, demonstrating downregulation of phosphorylation by ischemia-reperfusion, were grouped together in the other end of the heatmap (Fig. 2b).

### ROR1 is expressed in healthy and ischemic pig heart

**Fig. 3:**
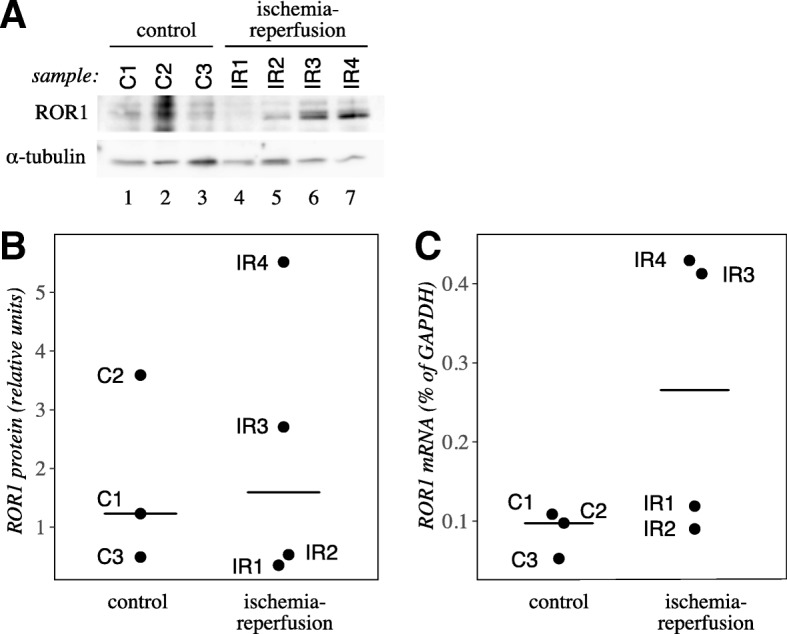
ROR1 expression in adult pig heart. **a** Western analysis of ROR1 expression in pig heart samples. An approximately 130 kDa band, corresponding to the predicted size of ROR1, was detected. **b** Densitometric quantification of ROR1 protein level relative to α-tubulin. **c** *ROR1* mRNA expression relative to *GAPDH* mRNA expression in pig heart samples was measured by real-time RT-PCR. In B and C, values for individual samples (*n* = 3 for control, *n* = 4 for ischemia-reperfusion) are plotted. Medians are indicated with horizontal lines

As ROR1 was one of the most significantly regulated RTKs in the human heart expression analysis (Fig. 1b), and significantly downregulated in the ischemic pig heart (Fig. 2), it was selected for further validation. To confirm the presence of ROR1 in the heart in vivo, lysates from pig myocardium were analyzed by Western blotting with a polyclonal anti-ROR1 antibody. A band of the predicted size for full-length ROR1 protein (130 kDa) was detected in both control and ischemia-reperfusion-injured samples (Fig. 3a and b). The expression of *ROR1* mRNA was confirmed by RT-PCR (Fig. 3c). In both analyses, ROR1 expression did not demonstrate statistically significant difference between control and ischemia-reperfusion samples.

### ROR1 expression and phosphorylation are downregulated by hypoxia-reoxygenation in cardiomyocytes in vitro

Analyses of the samples available from the IST Online database (Fig. 1) and the pig in vivo model (Figs. 2 and 3) indicated the presence of ROR1 in the heart tissue after ischemia and reperfusion, but did not allow for temporal analyses of ROR1 expression or activity at different time points after hypoxia and reoxygenation. To address the ROR1 regulation in vitro in cardiomyocytes under conditions simulating ischemia-reperfusion, HL-1 mouse atrial cardiomyocytes and H9c2 rat cardiomyoblasts were exposed to hypoxia (1% O_2_) in a hypoxic workstation for 1, 3 or 24 h. Cells were subsequently either returned to normoxia for 3 or 24 h (hypoxia-reoxygenation), or directly lysed for expression analysis (hypoxia alone). ROR1 protein expression was analyzed by Western blotting.

**Fig. 4:**
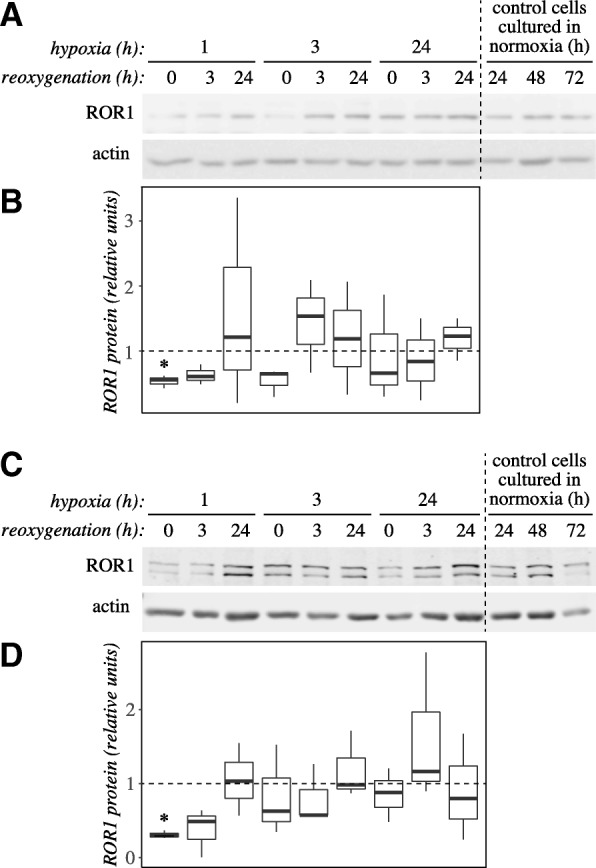
ROR1 protein level is regulated in cardiomyocytes in response to hypoxia and reoxygenation*.* **a** A representative Western analysis of ROR1 protein level in HL-1 cardiomyocytes after treatment with hypoxia and reoxygenation. Cells were allowed to adhere for 24 h after plating in normoxia. This was followed by culturing the cells in a hypoxic work station at 1% O_2_ (hypoxia) and subsequently again in the regular cell incubator in normoxia (reoxygenation) for the indicated periods of time. As time points were distributed over three days after plating, control samples cultured in normoxia for 24, 48 or 72 h were also analyzed. Time points (hypoxia+reoxygenation) 1 + 0, 1 + 3, 3 + 0 and 3 + 3 are comparable to the 24 h control (lane 10), time points 1 + 24, 3 + 24, 24 + 0 and 24 + 3 to the 48 h control (lane 11), and time points 24 + 24 to the 72 h control (lane 12). **b** A box plot presentation of quantitation of ROR1 bands from three Western blots similar to the one shown in panel A. ROR1 band intensities were normalized to each sample’s actin level, and subsequently divided by the control sample value of the respective timepoint. **c** A representative Western analysis of ROR1 protein levels in H9c2 cardiomyoblasts after treatment with hypoxia and reoxygenation. Experiment was carried out as shown for HL-1 cells in panel A. The antibody recognized two bands between 130 and 180 kDa, that both were down-regulated by ROR1 siRNA knockdown (data not shown). **d** A box plot presentation of quantitation of ROR1 bands from three Western blots similar to the one shown in panel C. Data were normalized as for panel B. Asterisk indicates significant difference in expression (*P* < 0.05) as compared to control samples

In HL-1 cells, the total ROR1 protein level was significantly reduced by 1 h treatment in hypoxia (*P* = 0.014), remained low 3 h after reoxygenation, and was recovered with a high variation in expression after 24 h of reoxygenation (Fig. 4a and b). Treatment for 3 h in hypoxia also initially slightly reduced ROR1 protein expression, but the expression recovered already 3 h after reoxygenation (Fig. 4a and b). The longest time point of hypoxia analyzed, 24 h, did not result in changes in ROR1 protein levels (Fig. 4a and b). In H9c2 cells, downregulation of ROR1 was similarly observed in response to treatment for 1 h in hypoxia (*P* = 0.002), followed by a trend of expression returning back to the level of control samples after reoxygenation (Fig. 4c and d). In contrast to ROR1, ROR2 expression was not significantly regulated by hypoxia-reoxygenation (Additional file 5 A and B).

To address whether ROR1 phosphorylation was regulated in response to hypoxia, ROR1 was immunoprecipitated from HL-1 cell lysates followed by anti-phosphotyrosine Western analysis. The samples were collected at two time points: after one hour of hypoxia, a time point that demonstrated significant downregulation of total ROR1 protein level in both HL-1 and H9c2 cells (Fig. 4), and after one hour of hypoxia followed by 24 h of reoxygenation, a schedule chosen to reflect the ischemia-reperfusion treatment used for the in vivo analysis (Fig. 2). ROR1 phosphorylation was downregulated in response to both treatments (Additional file 6), suggesting that hypoxia alone is sufficient for the observed effect.

### ROR1 knockdown increases the viability of cardiomyocytes in vitro

**Fig. 5:**
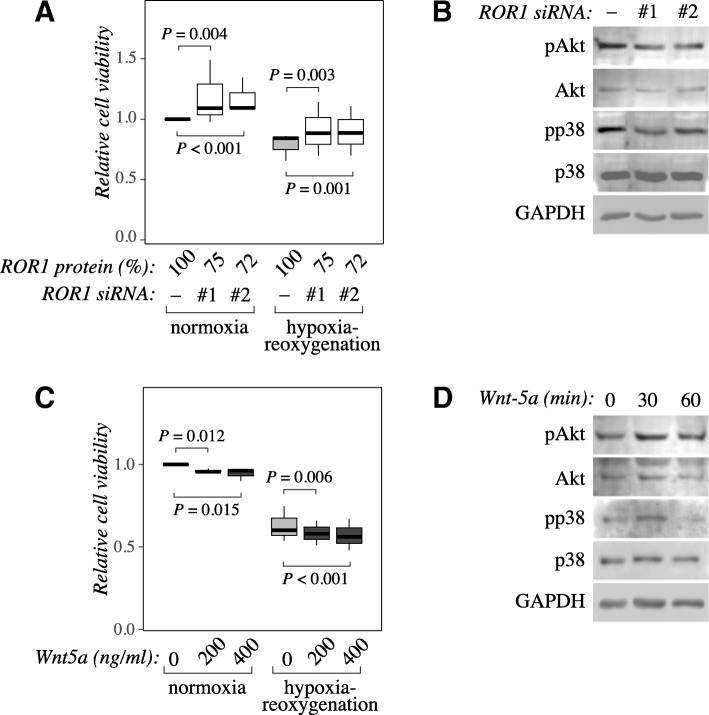
ROR1 knockdown reduces but Wnt-5a ligand treatment increases cardiomyocyte viability under normoxia and hypoxia-reoxygenation*.* **a** HL-1 cells were transfected with two different siRNAs targeting ROR1 (*ROR1* siRNA #1 and #2) or negative control siRNA. Twenty-four hours after transfection, cells were either transferred into a hypoxic work station (1% O_2_) or were maintained in normoxia as controls. After another 24 h, all cells were returned to normoxia for 24 h to allow for reoxygenation. Cell viability was analyzed using the MTT assay. A box plot presentation is shown indicating cell viability as normalized to negative control siRNA-treated cells cultured in normoxia. The efficacy of the *ROR1* siRNAs in down-regulating ROR1 expression is indicated (ROR1 protein %). Three independent experiments each including six replicates were carried out. **b** Western analysis of total and phosphorylated Akt and p38 in HL-1 cells lysed 48 h after siRNA transfection. **c** HL-1 cells were treated with the indicated concentrations of Wnt-5a since plating. Twenty-four hours after plating, cells were either transferred into a hypoxic work station (1% O_2_) or were maintained in normoxia as controls. After another 48 h, all cells were returned to normoxia for 24 h to allow for reoxygenation. Cell viability was analyzed using the MTT assay. A box plot presentation is shown indicating cell viability as normalized to cells cultured in the absence of the ligand in normoxia. Three independent experiments each including three replicates were carried out. **d** Western analysis of total and phosphorylated Akt and p38 in HL-1 cells treated or not with 400 ng/ml of Wnt-5a for 30 or 60 min. Negative control cells were lysed at the same time as the 30-min sample

The regulation of ROR1 expression in both ischemia-reperfusion and hypoxia-reoxygenation models indicated functional relevance for ROR1 activity in the hypoxic heart. To address the contribution of ROR1 for the cardiomyocyte viability, expression of the receptor was knocked down in HL-1 cells by RNA interference, and the relative amount of viable cells was measured by MTT assay. Two *ROR1*-targeting siRNAs significantly enhanced the cellular viability both in normoxia (*P* = 0.004 and *P* < 0.001 for siRNAs #1 and #2, respectively; *n* = 18) and after 24 h of hypoxia followed by 24 h of reoxygenation (*P* = 0.003 and *P* = 0.001 for siRNAs #1 and #2, respectively; n = 18), when compared to negative control siRNAs (Fig. 5a and Additional file 5 E). Consistent with the expression data, knockdown of ROR2 expression did not significantly affect HL-1 cell viability (Additional file 5 C-E), suggesting that the effect was specific for ROR1 within the ROR subfamily of RTKs.

### ROR1 promotes phosphorylation of Akt and p38

To study the downstream signaling effects of ROR1 knockdown, phosphorylation status of Akt and p38 was studied by Western blotting, as these pathways have been shown to be involved in ROR1 signaling [25, 26]. Phosphorylation of both Akt and p38 was decreased in HL-1 cells 24 h after siRNA transfection (Fig. 5b).

Wnt-5a is an activating ligand of ROR1 [27, 28] in addition to other receptor such as ROR2 [27–29], RYK [30] and Frizzled receptors [31]. Stimulation of HL-1 cells for 96 h with 200 or 400 ng/ml Wnt-5a significantly reduced the viability of the cells in both normoxia (*P* = 0.012 and *P* = 0.015, for 200 and 400 ng/ml, respectively; *n* = 9) and after 48-h hypoxia followed by 24 h of reoxygenation (*P* = 0.006 and *P* < 0.001, for 200 and 400 ng/ml, respectively; n = 9), as compared to non-treated controls (Fig. 5c). In accordance with the decrease in the level of phosphorylated Akt and p38 by ROR1-targeted RNA interference, phosphorylation of both Akt and p38 was enhanced in response to treatment with 400 ng/ml of Wnt-5a for 30 min (Fig. 5d).

Taken together, these results indicate that ROR1 expression and activity suppress cardiomyocyte viability in vitro, and that the pathways involved include Akt and p38.

## Discussion

To address the potential of RTKs as therapeutic targets in myocardial ischemia, expression and phosphorylation of RTKs was systematically analyzed in human and porcine ischemic heart samples. A subgroup of RTKs, both present in phosphorylated form in the ischemic myocardium, and differentially regulated at the expression level between the ischemic and normoxic samples, was identified. This subgroup included ALK, AXL, EGFR, EPHB2, ERBB2, FGFR2, KIT, ROR1, ROR2 and TIE1. As the role of ROR1 in ischemic heart has not previously been addressed, it was selected for further analyses. ROR1 expression and phosphorylation were found to be downregulated in cardiomyocytes in response to hypoxia. Moreover, functional in vitro experiments with RNA interference and Wnt-5a stimulation indicated that targeting of ROR1 enhances cardiomyocyte viability.

Analysis of RTK expression in the acute myocardial infarction samples revealed 4 RTKs with significantly reduced expression (*EGFR*, *ERBB2*, *ERBB3* and *EPHA2*) when compared to healthy heart. Of these, *ERBB2* has also previously been shown to be downregulated in hypoxic human heart [8]. While the other two ERBB family members, *EGFR* and *ERBB3*, were down-regulated in our analyses, the previous report by Munk et al. [8] indicates upregulation of *EGFR* and no change for *ERBB3* expression in hypoxia. Interestingly, EPHA2 has been shown to have cardioprotective potential in mouse models of myocardial ischemia and ischemic cardiomyopathy [32, 33].

Analysis of RTK expression in the ischemic cardiomyopathy samples revealed 3 RTKs with significantly enhanced expression (*ROR1, KIT* and *TIE1*) and 11 with significantly reduced expression (*EPHA2, LTK, PDGFRB, ERBB3, FGFR2, AXL, ALK, ROR2, EPHA8, EPHB2* and *EPHB4*) when compared to healthy heart. *ROR1* expression was most significantly upregulated. However, the roles for most RTKs identified in our analyses in ischemic cardiomyopathy remain to be elucidated in future studies.

The phosphoarray analysis of porcine myocardial samples indicated the highest phosphorylation level for EGFR, ROR2, INSR, EPHB3, EPHB2, TEK, RYK, ROR1 and EPHB6. For ROR1 and VEGFR2, difference in phosphorylation between control and ischemia-reperfusion samples was statistically significant. While details about the antibodies included in the phospho-RTK array are not publicly available, the kit has been designed to detect human receptors. A conservation analysis between the human and porcine RTK protein sequences indicated high conservation for most receptors (median global similarity = 93.1%) and global similarity of 92.4% for ROR1 (Additional file 7). However, the specificity of the antibodies in the array could not be directly controlled and, especially, the phosphorylation status of ROR1 in ischemic heart remains to be studied in future analyses. Nevertheless, a reproducible set of active RTKs in the ischemic heart was detected, including both novel receptors (e.g. ROR1, ROR2) and ones formerly known to be active in the heart (EGFR, ERBB2, INSR, VEGFR2).

Both ROR1 expression and phosphorylation were found to be down-regulated in cardiomyocytes in vitro. Although ROR1 phosphorylation was downregulated also in the pig model of acute ischemia-reperfusion injury in vivo, *ROR1* expression in the pig model demonstrated a nonsignificant trend for increase. Moreover, *ROR1* mRNA expression was upregulated in the ischemic cardiomyopathy samples. These findings could reflect the intrinsic differences in measuring protein phosphorylation vs. expression, and the associated feed-back regulation, but also the duration of hypoxia in the different models. For example, ischemic cardiomyopathy is a chronic ischemic disease involving heart failure, while the ischemia-reperfusion-model in the pig and the in vitro analyses are models for more acute hypoxic conditions.

ROR1 signaling was demonstrated to inhibit cardiomyocyte survival, as its knockdown increased, while its ligand activation decreased, cellular viability. ROR1 and ROR2 comprise the Receptor tyrosine kinase-like Orphan Receptor (ROR) family. While ROR signaling in the ischemic heart has not previously been addressed, both receptors are known to regulate heart development during mouse embryogenesis [6, 7, 34]. Interestingly, in our in vitro analyses, hypoxia affected expression of ROR1, but not of ROR2, and knockdown of *ROR1*, but not of *ROR2*, enhanced cardiomyocyte viability, implying that the two receptors have overlapping but not fully redundant biological functions in these cells. The pathways regulating ROR1 functions in cardiomyocytes may involve p38, as our phospho-Western analyses indicated p38 regulation both in response to *ROR1* knockdown as well as to Wnt-5a ligand stimulation. Indeed, inhibition of p38 signaling has been shown to enable adult cardiomyocyte proliferation [35] and promote cardiac regeneration [36]. Interestingly, a ROR1-targeting monoclonal antibody, cirmtuzumab, has been developed for the treatment of chronic lymphatic leukemia [37] allowing future analysis of its effect on normal and ischemic heart.

## Conclusions

In conclusion, we describe an RTK-proteome level approach to characterize RTK expression and activity in the ischemic heart. ROR1 was identified as one of the RTKs that was both present in the ischemic heart in an active form and regulated in expression in a manner that associated with clinical and experimental ischemia. Manipulation of ROR1 expression and activity in vitro indicated a functional role for ROR1 in suppressing cardiomyocyte viability. These findings warrant further studies addressing the targeting of ROR1 as an approach to treat ischemic heart disease.

## Additional files

Additional file 1: RTKs included in in silico analysis of expression from IST Online data. Statistical analyses of differences in RTK mRNA expression between samples representing healthy human heart and ischemic cardiomyopathy or acute myocardial infarction. (XLSX 15 kb)

Additional file 2: Phospho-RTK array of ischemia-reperfusion-injured pig hearts. A) Representative phospho-RTK array blots from control and ischemia-reperfusion-injured pig heart samples. B) Array overlay and a corresponding coordinate table indicating the location of RTKs in the array. Each of the 49 RTKs included in the array are represented by two adjacent dots. (PDF 229 kb)

Additional file 3: Heatmaps of RTKs demonstrating significant changes in mRNA expression in either ischemic cardiomyopathy or acute myocardial infarction when compared to healthy heart. RTKs with significant expression level differences (FDR-corrected *P* values < 0.05) between pairwise group comparisons were selected for visualization. A) Ischemic cardiomyopathy vs. healthy heart. B) Acute myocardial infarction vs. healthy heart. The data represent normalized log2-transformed Affymetrix gene expression values from the IST Online database. (PDF 321 kb)

Additional file 4: Myocardial damage in ischemia-reperfusion-injured pig hearts. A) Plasma troponin T levels from four ischemia-reperfusion-injured pigs were collected at baseline, and 6 and 24 h after reperfusion. Medians are indicated with horizontal lines. B) Representative HE-stained images from a healthy and ischemia-reperfusion-injured pig heart (sample collected 31 h after reperfusion). (PDF 44885 kb)

Additional file 5: ROR2 in cardiomyocytes. A) A representative Western analysis of ROR2 protein level in HL-1 cardiomyocytes after treatment with hypoxia and reoxygenation. All cells were first allowed to adhere for 24 h after plating in normoxic conditions. This was followed by culturing the cells in a hypoxic work station at 1% O_2_ (hypoxia) and subsequently again in the regular cell incubator in normoxia (reoxygenation) for the indicated periods of time. As different time points were distributed over three days after plating, control samples cultured in normoxia for 24, 48 or 72 h were also analyzed. Time points (hypoxia+reoxygenation) 1 + 0, 1 + 3, 3 + 0 and 3 + 3 are comparable to the 24 h control (lane 10), time points 1 + 24, 3 + 24, 24 + 0 and 24 + 3 to the 48 h control (lane 11), and time points 24 + 24 to the 72 h control (lane 12). B) A box plot presentation of densitometric quantitation of ROR1 bands from three replicate Western blots similar to the one shown in panel A. ROR1 band intensities were first normalized to each sample’s actin level, and subsequently divided by the control sample value of the respective time point. C) Effect of ROR2 knockdown on cellular viability. HL-1 cells were transfected with two different siRNAs targeting ROR2 (ROR2 siRNA #1 and #2) or negative control siRNA. Twenty-four hours after transfection, cells were either transferred into a hypoxic work station (1% O_2_) or were maintained in normoxia as controls. After another 24 h, all cells were returned to normoxia for 24 h to allow for reoxygenation. Cell viability was analyzed using the MTT assay. A box plot presentation is shown indicating cell viability as normalized to negative control siRNA-treated cells cultured in normoxia. Three independent experiments each including six replicates were carried out. D) Western analysis of ROR2 protein expression after ROR2 siRNA treatments. E) Western analyses of ROR1 and ROR2 protein expression after ROR1 siRNA treatment. (PDF 3815 kb)

Additional file 6: Analysis of ROR1 phosphorylation in HL-1 cardiomyocytes after hypoxia and reoxygenation. A) Western analysis of tyrosine phosphorylation after ROR1 immunoprecipitation. Cells were first allowed to adhere for 24 h after plating in normoxic conditions. This was followed by culturing the cells in a hypoxic work station at 1% O_2_ (hypoxia) and subsequently again in the regular cell incubator in normoxia (reoxygenation) for the indicated periods of time. As different time points were distributed over two days after plating, control samples cultured in normoxia for 24 or 48 h were also analyzed. Time point of one hour of hypoxia (lane 1) is comparable to the 24 h control (lane 2) and time point of one hour of hypoxia and 24 h of reoxygenation (lane 3) is comparable to the 48 h control (lane 4). B) Quantitation of ROR1 phosphorylation relative to total protein. (PDF 179 kb)

Additional file 7: RTK similarity and identity between pig and human. (XLSX 12 kb)

## Abbreviations

FDR: False discovery rate
ROR: Receptor tyrosine kinase-like Orphan Receptor
RTK: Receptor tyrosine kinase
