# Supplementary material for: Receptor tyrosine kinase profiling of ischemic heart identifies ROR1 as a potential therapeutic target
Source: BMC Cardiovasc Disord. 2018 Oct 20;18:196. doi: 10.1186/s12872-018-0933-y (PMC6196006; doi:10.1186/s12872-018-0933-y)
Supplement: Supplementary file 2 — Phospho-RTK array of ischemia-reperfusion-injured pig hearts. A) Representative phospho-RTK array blots from control and ischemia-reperfusion-injured pig heart samples. B) Array overlay and a corresponding coordinate table indicating the location of RTKs in the array. Each of the 49 RTKs included in the array are represented by two adjacent dots. (PDF 229 kb) [file 12872_2018_933_MOESM2_ESM.pdf]

A

normal heart *ischemia-reperfusion*

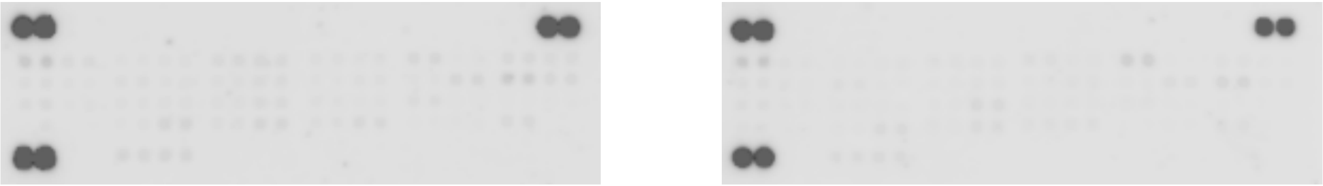

B

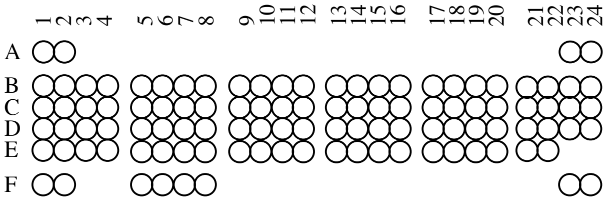

Coordinate	RTK	Coordinate	RTK
A1, A2	Reference spots	D1, D2	TEK
A23, A24	Reference spots	D3, D4	NTRK1
B1, B2	EGFR	D5, D6	NTRK2
B3, B4	ERBB2	D7, D8	NTRK3
B5, B6	ERBB3	D9, D10	VEGFR1
B7, B8	ERBB4	D11, D12	VEGFR2
B9, B10	FGFR1	D13, D14	VEGFR3
B11, B12	FGFR2	D15, D16	MUSK
B13, B14	FGFR3	D17, D18	EPHA1
B15, B16	FGFR4	D19, D20	EPHA2
B17, B18	INSR	D21, D22	EPHA3
B19, B20	IGF1R	D23, D24	EPHA4
B21, B22	AXL	E1, E2	EPHA6
B23, B24	TYRO3	E3, E4	EPHA7
C1, C2	MERTK	E5, E6	EPHB1
C3, C4	MET	E7, E8	EPHB2
C5, C6	MST1R	E9, E10	EPHB4
C7, C8	PDGFRA	E11, E12	EPHB6
C9, C10	PDGFRB	E13, E14	ALK
C11, C12	KIT	E15, E16	DDR1
C13, C14	FLT3	E17, E18	DDR2
C15, C16	CSFR	E19, E20	EPHA5
C17, C18	RET	E21, E22	EPHA10
C19, C20	ROR1	F1, F2	Reference spots
C21, C22	ROR2	F5, F6	EPHB3
C23, C24	TIE1	F7, F8	RYK
		F23, F24	Negative control

Additional file 2
