# Supplementary figures and images for: Receptor tyrosine kinase profiling of ischemic heart identifies ROR1 as a potential therapeutic target
Source: BMC Cardiovasc Disord. 2018 Oct 20;18:196. doi: 10.1186/s12872-018-0933-y (PMC6196006; doi:10.1186/s12872-018-0933-y)

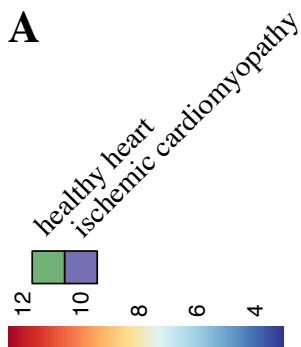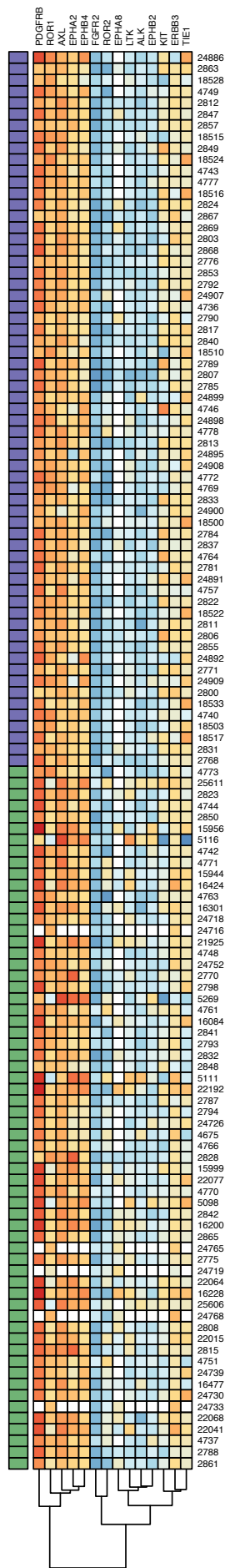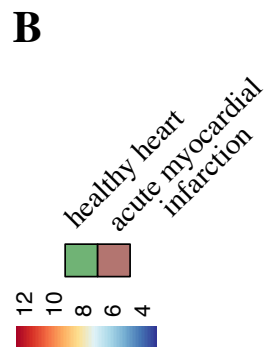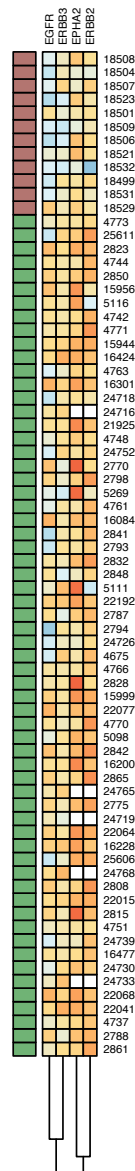

Additional file 3

Supplement: Supplementary file 3 — Heatmaps of RTKs demonstrating significant changes in mRNA expression in either ischemic cardiomyopathy or acute myocardial infarction when compared to healthy heart. RTKs with significant expression level differences (FDR-corrected P values < 0.05) between pairwise group comparisons were selected for visualization. A) Ischemic cardiomyopathy vs. healthy heart. B) Acute myocardial infarction vs. healthy heart. The data represent normalized log2-transformed Affymetrix gene expression values from the IST Online database. (PDF 321 kb) [file 12872_2018_933_MOESM3_ESM.pdf]

**A**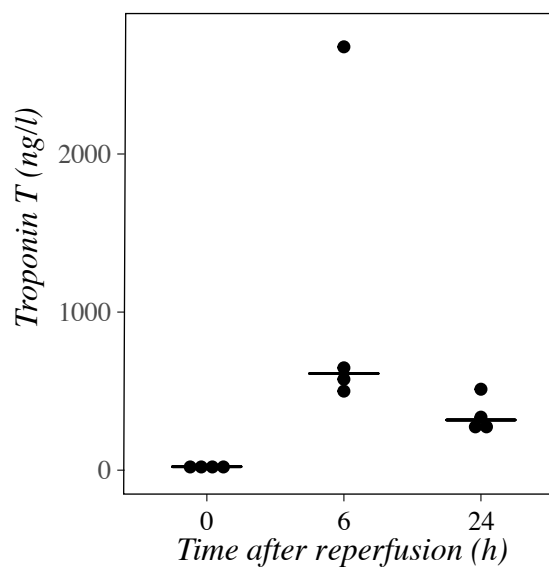**B**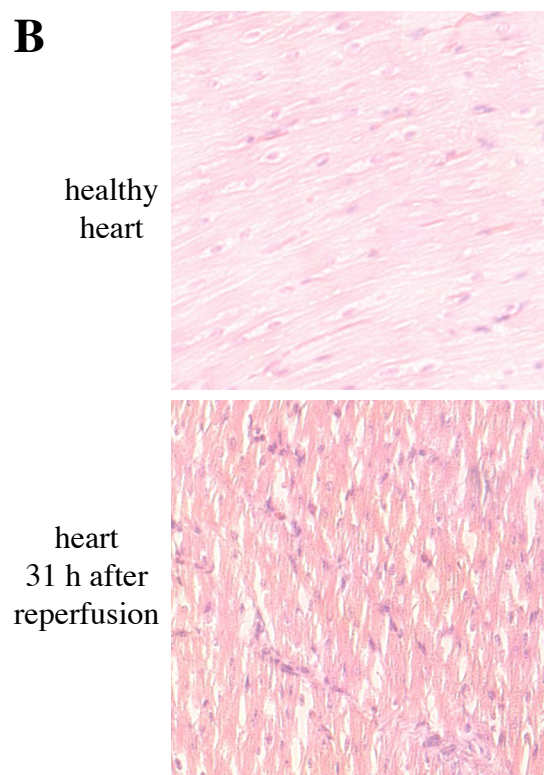**Additional file 4**

Supplement: Supplementary file 4 — Myocardial damage in ischemia-reperfusion-injured pig hearts. A) Plasma troponin T levels from four ischemia-reperfusion-injured pigs were collected at baseline, and 6 and 24 h after reperfusion. Medians are indicated with horizontal lines. B) Representative HE-stained images from a healthy and ischemia-reperfusion-injured pig heart (sample collected 31 h after reperfusion). (PDF 44885 kb) [file 12872_2018_933_MOESM4_ESM.pdf]

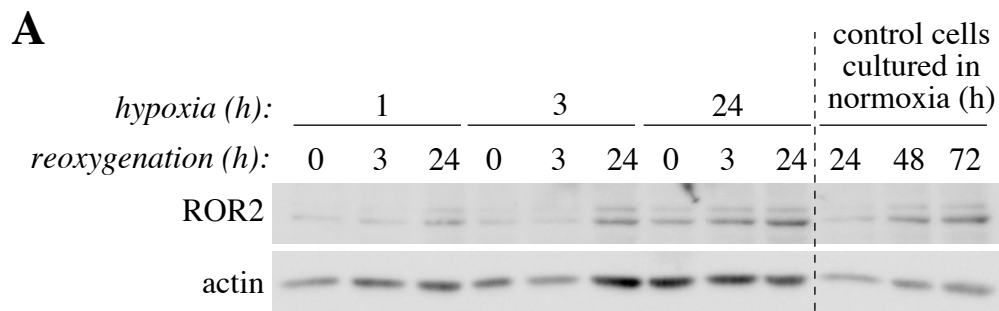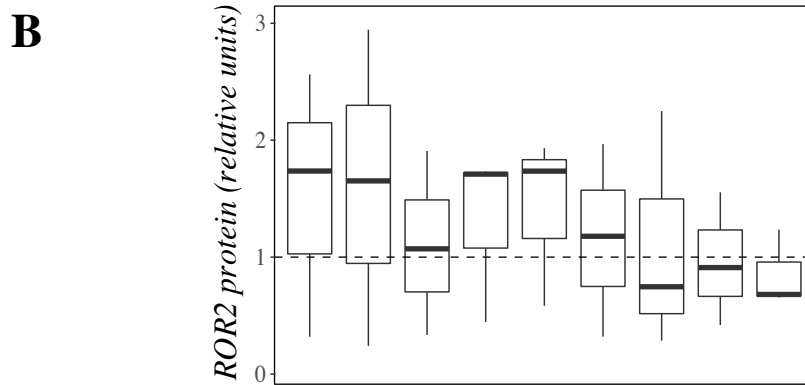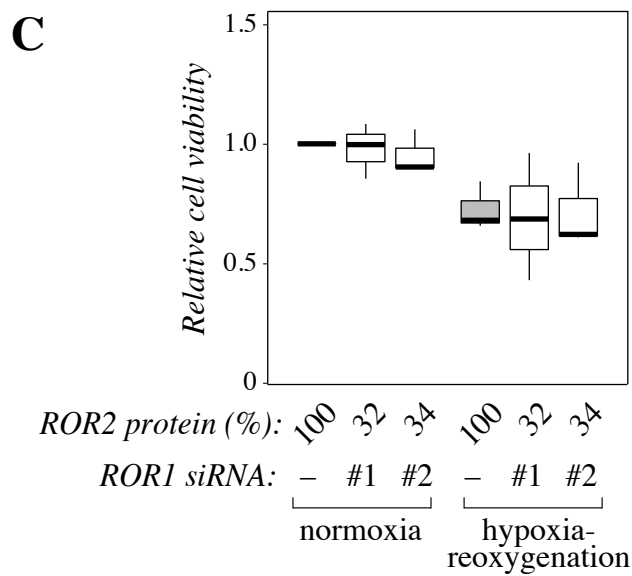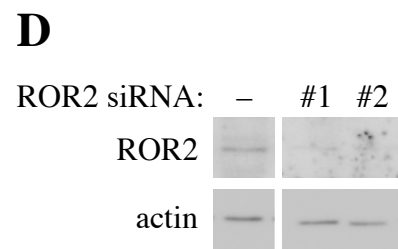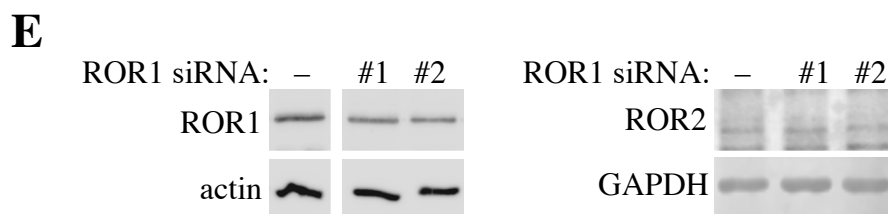

Supplement: Supplementary file 5 — ROR2 in cardiomyocytes. A) A representative Western analysis of ROR2 protein level in HL-1 cardiomyocytes after treatment with hypoxia and reoxygenation. All cells were first allowed to adhere for 24 h after plating in normoxic conditions. This was followed by culturing the cells in a hypoxic work station at 1% O2 (hypoxia) and subsequently again in the regular cell incubator in normoxia (reoxygenation) for the indicated periods of time. As different time points were distributed over three days after plating, control samples cultured in normoxia for 24, 48 or 72 h were also analyzed. Time points (hypoxia+reoxygenation) 1 + 0, 1 + 3, 3 + 0 and 3 + 3 are comparable to the 24 h control (lane 10), time points 1 + 24, 3 + 24, 24 + 0 and 24 + 3 to the 48 h control (lane 11), and time points 24 + 24 to the 72 h control (lane 12). B) A box plot presentation of densitometric quantitation of ROR1 bands from three replicate Western blots similar to the one shown in panel A. ROR1 band intensities were first normalized to each sample’s actin level, and subsequently divided by the control sample value of the respective time point. C) Effect of ROR2 knockdown on cellular viability. HL-1 cells were transfected with two different siRNAs targeting ROR2 (ROR2 siRNA #1 and #2) or negative control siRNA. Twenty-four hours after transfection, cells were either transferred into a hypoxic work station (1% O2) or were maintained in normoxia as controls. After another 24 h, all cells were returned to normoxia for 24 h to allow for reoxygenation. Cell viability was analyzed using the MTT assay. A box plot presentation is shown indicating cell viability as normalized to negative control siRNA-treated cells cultured in normoxia. Three independent experiments each including six replicates were carried out. D) Western analysis of ROR2 protein expression after ROR2 siRNA treatments. E) Western analyses of ROR1 and ROR2 protein expression after ROR1 siRNA treatment. (PDF 3815 kb) [file 12872_2018_933_MOESM5_ESM.pdf]

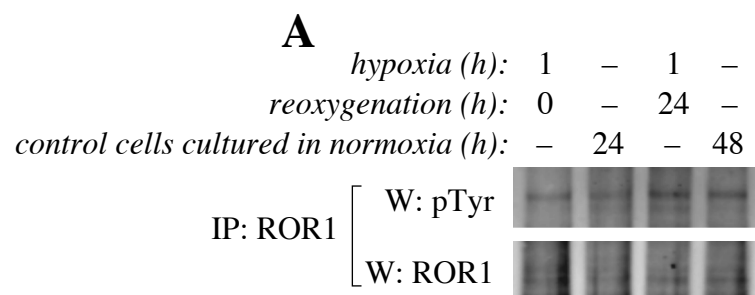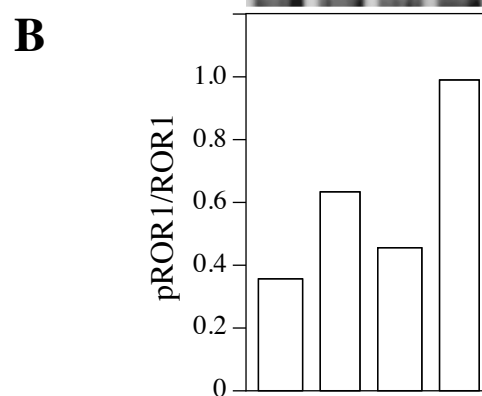

Supplement: Supplementary file 6 — Analysis of ROR1 phosphorylation in HL-1 cardiomyocytes after hypoxia and reoxygenation. A) Western analysis of tyrosine phosphorylation after ROR1 immunoprecipitation. Cells were first allowed to adhere for 24 h after plating in normoxic conditions. This was followed by culturing the cells in a hypoxic work station at 1% O2 (hypoxia) and subsequently again in the regular cell incubator in normoxia (reoxygenation) for the indicated periods of time. As different time points were distributed over two days after plating, control samples cultured in normoxia for 24 or 48 h were also analyzed. Time point of one hour of hypoxia (lane 1) is comparable to the 24 h control (lane 2) and time point of one hour of hypoxia and 24 h of reoxygenation (lane 3) is comparable to the 48 h control (lane 4). B) Quantitation of ROR1 phosphorylation relative to total protein. (PDF 179 kb) [file 12872_2018_933_MOESM6_ESM.pdf]
